# Sesquiterpene Lactones and Their Derivatives Inhibit High Glucose-Induced NF-κB Activation and MCP-1 and TGF-β1 Expression in Rat Mesangial Cells

**DOI:** 10.3390/molecules181013061

**Published:** 2013-10-21

**Authors:** Qian-Qian Jia, Jian-Cheng Wang, Jing Long, Yan Zhao, Si-Jia Chen, Jia-Dai Zhai, Lian-Bo Wei, Quan Zhang, Yue Chen, Hai-Bo Long

**Affiliations:** 1 Department of Nephrology, Zhujiang Hospital, Southern Medical University, Guangzhou 510000, China; E-Mails: xiaobing716@sina.com.cn (Q.-Q.J.); jason20013@163.com (J.-C.W.); yan.er0306@163.com (Y.Z); xxxholicsj@163.com (S.-J.C.); weilianbo@163.com (L.-B.W.); 2 College of Pharmacy, the State Key Laboratory of Elemento-Organic Chemistry, and Tianjin Key Laboratory of Molecular Drug Research, Nankai University, Tianjin 300071, China; E-Mails: jingtufei@163.com (J.L.); zhaijiadai@163.com (J.-D.Z.); zhangquan612@163.com (Q.Z.); yuechen@nankai.edu.cn (Y.C.)

**Keywords:** diabetic nephropathy, sesquiterpene lactone, NF-κB, deriviative, sythesis

## Abstract

Diabetic nephropathy (DN) is one of the most common and serious chronic complications of diabetes mellitus, however, no efficient clinical drugs exist for the treatment of DN. We selected and synthesized several sesquiterpene lactones (SLs), and then used the MTT assay to detect rat mesangial cells (MCs) proliferation, ELISA to measure the expression level of monocyte chemoattractant protein-1 (MCP-1), transforming growth factor beta (TGF-β1) and fibronectin(FN), real-time fluorescent quantitative PCR analysis to measure the MCP-1 and TGF-β1 gene expression, western blot to detect the level of IκBα protein and EMSA to measure the activation of nuclear factor kappa B (NF-κB). We discovered that SLs, including parthenolide (PTL), micheliolide (MCL), arglabin, and isoalantolactone (IAL), as well as several synthetic analogs of these molecules, could effectively attenuate the high glucose-stimulated activation of NF-κB, the degradation of IκBα, and the expression of MCP-1, TGF-β1 and FN in rat mesangial cells (MCs). These findings suggest that SLs and their derivatives have potential as candidate drugs for the treatment of DN.

## 1. Introduction

In many Western countries, diabetes mellitus (DM) is the third largest chronic non-communicable disease, preceded only by cardiovascular disease and cancer. Diabetic nephropathy (DN), characterized by the angiopathy of capillaries in the kidney glomeruli, is one of the most common and serious chronic complications of DM. DN can cause chronic kidney failure and end-stage kidney disease and is the leading cause of premature death in young diabetic patients (those between 50 and 70 years old). Combination therapy with ACE inhibitor drugs and angiotensin receptor blockers (ARBs) drugs may slow the progression of DN [1], however, according to Ongoing Telmisartan Alone and in Combination with Ramipril Global Endpoint Trial (ONTARGET), combination therapy may worsen major renal outcomes and may result in a decline in the glomerular filtration rate [2]. Interestingly, silymarin (milk thistle extract), an herb containing a mixture of anti-inflammatory components, has recently been shown to be effective in reducing proteinuria in patients with DN [3].

The pathogenesis of DM and the factors that contribute to this disease are complicated, although hyperglycemia and inflammation are known to be central to the initiation and pathogenesis of DN [4]. Inflammation and pro-inflammatory cytokines are known to play decisive roles in the development of DM-related microvascular complications. Nuclear factor kappa B (NF-κB) is an important regulator of the expression of inflammation and pro-inflammatory response genes such as monocyte chemoattractant protein-1 (MCP-1) and transforming growth factor beta (TGF-β1), which are secreted in the glomerular mesangial cells have become known as the chief mediators of the inflammation process in DN patients [5,6]. The activation of NF-κB can promote the expression of chemokine MCP-1, TGF-β1, as well as the extracellular matrix (ECM) proteins fibronectin (FN), leading to glomerular and tubulointerstitial leukocyte infiltration, glomerular mesangial matrix accumulation, and tubulointerstitial sclerosis [7,8]. Other studies have confirmed that the NF-κB inhibitor PDTC can block MCP-1 at both gene and protein levels. The expression of MCP-1 that is induced by the non-enzymatic glycosylation of albumin in human mesangial cells *in vitro*, suggesting that the occurrence of NF-κB-mediated DN is caused by the high-glucose-induced activation of NF-κB occurring through the protein kinase C-NF-κB pathway [7,8]. Regardless of duration of the disease process, DM patients have been found to have abnormal expression of NF-κB *in vivo*, suggesting that NF-κB is an important pathogenic factor in the occurrence and development of DN [9,10,11], therefore, the inhibition of NF-κB might have a substantial protective effect in DN, and the NF-κB signaling pathway, including MCP-1, TGF-β1 and FN, might be an important target to the treatment of this disorder [12,13].

MCP-1 is a β cytokine that is a member of the chemokine family and the chemokine-like factor superfamily and plays a critical role in the inflammatory response. Recent studies have shown that the excessive expression of MCP-1 is one of the main contributing factors for DN [14]. A study by Chow confirmed that MCP-1 is a strong stimulator of macrophage recruitment and that MCP-1 is increased in diabetic kidneys, indicating that the inflammation of kidneys in DM may be MCP-1-dependent [15]. TGF-β1 is recognized as another important factor in the pathogenesis of DN by mediating inflammatory response, which aggravates ECM including FN and collagen accumulation, as well as accelerates glomerularbrosis in diabetes [16]. NF-κB is reportedly involved in the oxidized low-density lipoprotein-mediated increment of TGF-β1 transcription [17].

NF-κB plays an important role in the signal transmission and a variety of gene expression in the nucleus. Inhibitor kappa B (IκBα) is a key protein of the transcription factor NF-κB. NF-κB remains in the cytoplasm in an inactive state under normal circumstances, bound by IκBα protein to form a complex. Immune stimuli can induce the phosphorylation and subsequent degradation of IκBα by the ubiquitin-proteasome pathway. After the depolymerization of NF-κB and IκBα, the nuclear localization sequences of the p50 and p65 subunits of NF-κB become exposed, and the activated NF-κB then enters the nucleus, binds to the DNA, and regulates the transcription of inflammatory genes; this process is important in mediating the inflammation and immune reactions [18,19,20]. Because IκBα is an inhibitor of NF-κB, the degradation of IκBα is an important indicator of NF-κB activation [21,22]. It was reported that hyperglycemia and advanced glycation end products are responsible for the activation of NF-κB.

**Figure 1:**
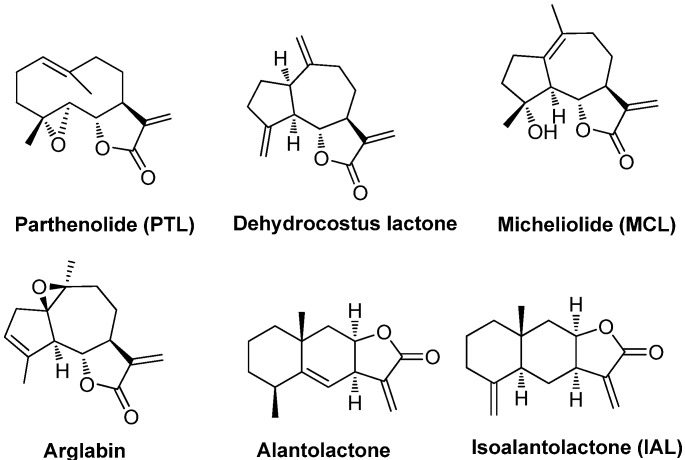
Structures of some natural sesquiterpene lactones.

Sesquiterpene lactones (SLs) are a large group of natural compounds with a wide range of biological activities, including anticancer and anti-inflammatory properties. SLs are primarily classified into the following groups: pseudoguainolides, guaianalides, germanocranolides, eudesmanolides, heliangolides, and hyptocretenolides [23]. Parthenolide (PTL, Figure 1), a prominent member of the germanocranolides, is the major active component in feverfew (*Tanacetum parthenium*), which is an herbal medicine that has been used for centuries in Western countries to treat migraines and rheumatoid arthritis [24]. Recent studies have demonstrated that PTL can inhibit inflammatory factors in human renal mesangial cells under hyperglycemic conditions [25], and that it may suppress tumor growth in a xenograft model of renal cell carcinoma by inhibiting the activation of NF-κB [26]. A study by Li showed that PTL could inhibit the high-glucose-induced expression of the inflammatory cytokines TNF-α, TGF-β1, IL-1β, IL-18, MIP-1α, and MCP-1 in human mesangial cells [25].

Dehydrocostus lactone, a natural guaianolide sesquiterpene lactone [27], was isolated from the roots of the traditional Chinese herb *Saussurea lappa* [28]. Dehydrocostus lactone has been shown to have many biological activities, including anti-inflammatory properties [29], however, this compound is not stable due to its rapid polymerization [30]. Arglabin, another member of the guaianolides, shows promising cytotoxicity against various tumor cell lines [31]. Arglabin has been modified to a water-soluble form (arglabin-DMA), which is a registered antitumor substance in the Republic of Kazakstan for the treatment of breast, colon, ovarian, and lung cancers [32]. Micheliolide (MCL), a naturally occurring guaianolide, has been isolated from *Michelia compressa* [33] and *Michelia champaca* [34]. We recently reported that MCL can selectively eradicate AML stem and progenitor cells and that its water-soluble form, DMAMCL, demonstrates excellent efficacy in the treatment of acute leukemia in mouse models [35]. Isoalantolactone (IAL) and alantolactone are the main active ingredients in the Chinese herb *Tumuxiang*, and IAL possesses a significant anti-inflammatory activity similar to that of silymarin [36].

The water-soluble forms of PTL and MCL (*i.e.*, DMAPT and DMAMCL) are anti-cancer drug candidates that are currently being tested in a clinical trial and a preclinical study, respectively, and both DMAPT and DMAMCL were found to have very low side toxicities [37]. Arglabin-DMA is in clinical application as an anti-cancer drug [32]. These facts suggest that SLs are generally “drug-like” compounds; therefore, drug discovery and development studies that are based on the SL backbone may have a high potential to identify drug candidates for the treatment of DN.

We examined whether any naturally-occurring SLs or artificial SL analogs can be considered as drug candidates for the treatment of DN. In this study, several natural SLs were chosen and were further modified to generate analogs that are potentially more stable. Each of the resulting compounds was then evaluated, at both the protein and cell levels, for the ability to affect DN.

## 2. Results and Discussion

### 2.1. Chemistry

**Scheme 1:**
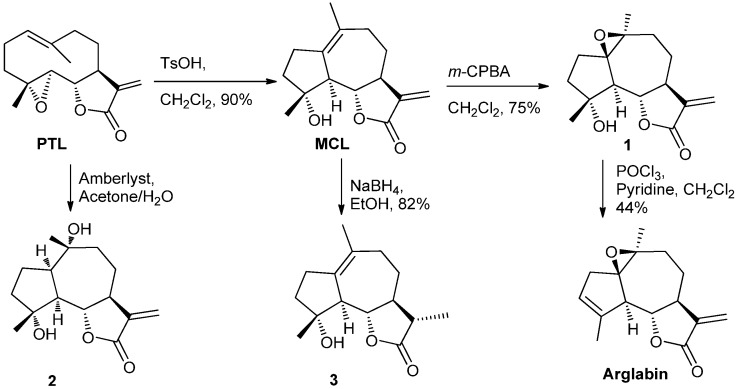
Syntheses of compounds MCL, Arglabin, **1**, **2**, and **3** started with parthenolide.

The syntheses of SLs was initiated with the readily available PTL (Scheme 1) [38]. PTL was treated with *p*-toluenesulfonic acid (TsOH) to obtain MCL and was then subjected to epoxidation with *m*-CPBA to afford compound **1**. This compound was then subjected to elimination with POCl_3_/pyridine to supply arglabin [38]. PTL was treated under aqueous conditions to generate compound **2**, and MCL was reduced to provide compound **3** [35].

**Scheme 2:**
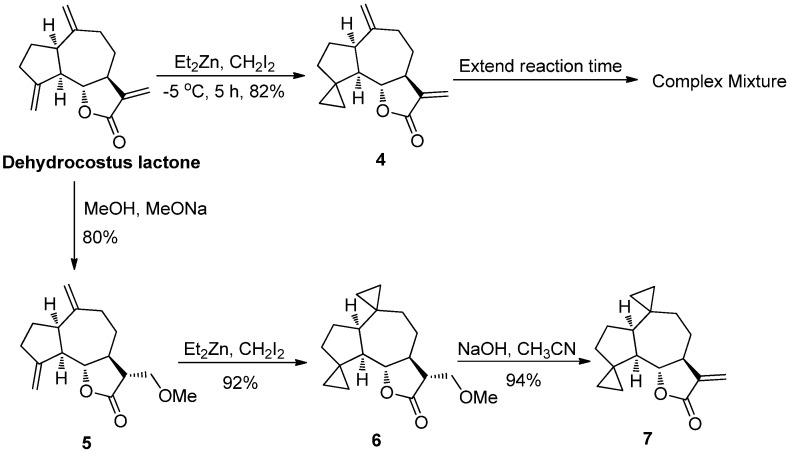
Synthesis of di-spirocyclopropyl analogue **7**.

Dehydrocostus lactone was treated with a standard Simmons-Smith reaction [39], and the resulting 4,14-mono-spirocyclopropyl compound (compound **4**) was obtained in an 82% yield (Scheme 2). However, compound **4** underwent an undesirable rapid polymerization, and attempts to extend the reaction time to install an additional cyclopropyl to generate compound **4** led to the formation of a complex mixture. Therefore, the 11,13-conjugated double bond of the dehydrocostus lactone compound was protected as a Michael adduct (compound **5**) [40], and compound **5** was then converted to compound **6** under Simmons-Smith reaction condition. Finally, the conjugated double bond was recovered by elimination to afford the target compound **7** [41].

**Scheme 3:**
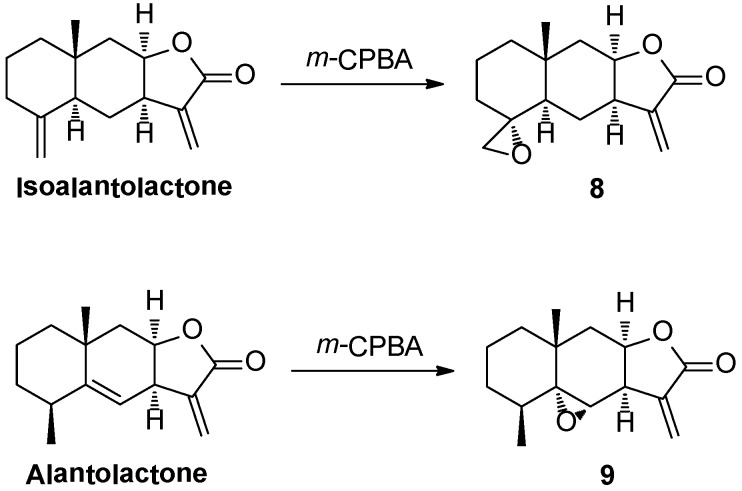
Synthesis of compounds **8** and **9**.

Compounds **8** and **9** were obtained by the epoxidation of IAL and alantolactone, respectively, with *m*-CPBA (Scheme 3) following a reported procedure [42].

### 2.2. Bioactivities

**Figure 2:**
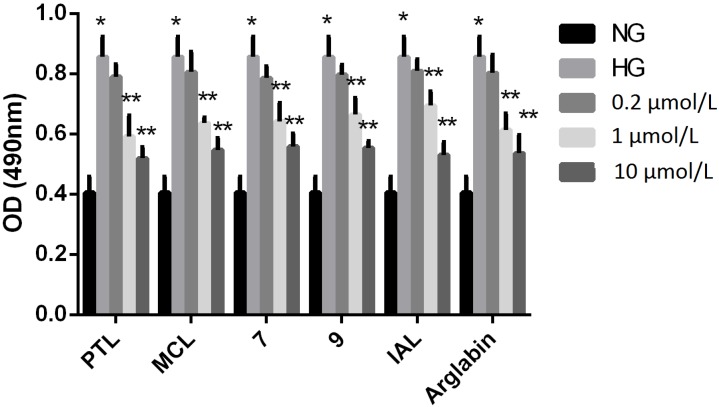
Effect of sesquiterpene lactones on HG-induced regulation of proliferation in MCs at a concentration of 0.2, 1, 10 μM for 24 h. NG: normal glucose; HG: high glucose. * *p* < 0.05 *vs.* NG, ** *p* < 0.05 *vs.* HG.

The proliferation of MCs was enhanced after 24 h in the presence of a high concentration of glucose (HG *vs.* NG, Figure 2). After treatment with 0.2, 1, 10 μM of the SLs, PTL, MCL, IAL, arglabin, and compounds **7**, and **9**, which were shown as white amporous powder, the cell proliferation rates in the groups treated with PTL, MCL, IAL, arglabin, compound **7**, and compound **9** were significantly lower than those of the HG-treated groups in a dose-dependent manner. Notably, in the groups treated with SLs at a concentration of 10 μM, the level of cell proliferation was comparable to that of the NG group, indicating that PTL and MCL were potent when applied at a concentration of 10 μM.

**Figure 3:**
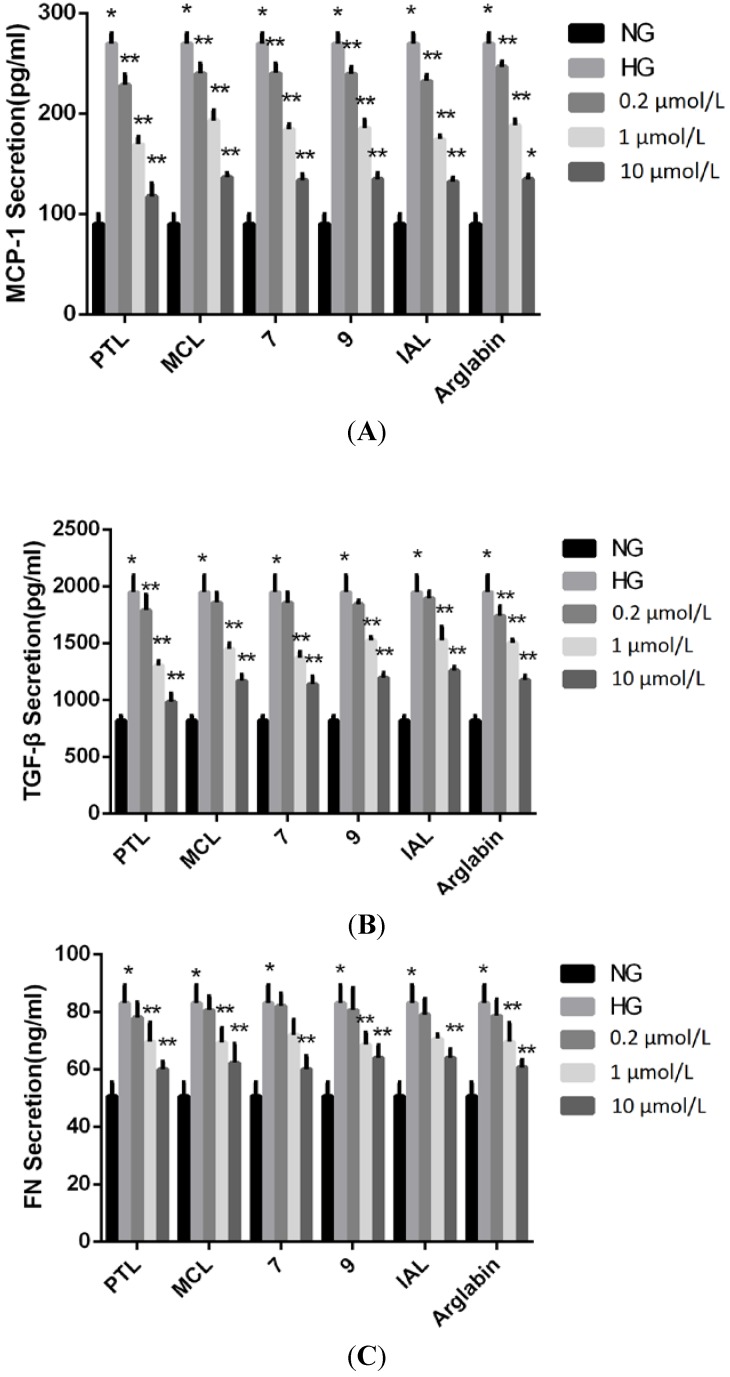
Effect of sesquiterpene lactones on HG-induced regulation of MCP-1, TGF-β1 and FN in MCs at a concentration of 0.2, 1, 10 μM. NG: normal glucose; HG: high glucose. * *p* < 0.05 *vs.* NG, ** *p* < 0.05 *vs.* HG.

The expression levels of MCP-1, TGF-β1 and FN in the cell culture supernatants of the HG-treated MCs were remarkably up-regulated compared to those of the NG-treated cells (Figure 3). Importantly, treatment with 1 and 10 μM of PTL, MCL, IAL, arglabin, compound **7**, or compound **9** significantly decreased the HG-induced production of MCP-1, TGF-β1 and FN. In addition, the expression levels of MCP-1, TGF-β1 and FN in the groups treated with PTL, MCL, IAL, Arglabin, compound **7** and compound **9** were essentially active at a concentration of 10 μM than the other concentration.

**Figure 4:**
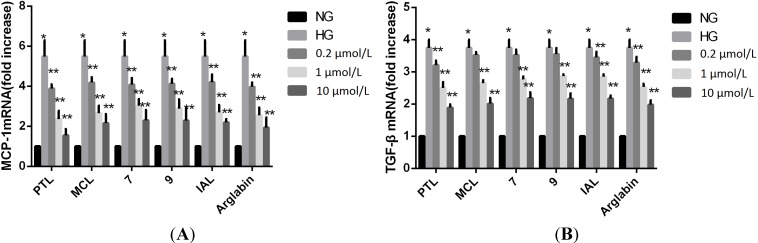
Effect of sesquiterpene lactones on HG-induced regulation of MCP-1 and TGF-β1 mRNA in MCs at a concentration of 0.2, 1, 10 μM. NG: normal glucose; HG: high glucose. * *p* < 0.05 *vs.* NG, ** *p* < 0.05 *vs.* HG.

The real-time fluorescent quantitative PCR detection results showed that the mRNA levels of MCP-1 and TGF-β1 in the HG-treated groups were elevated compared to those of the NG groups (*p* < 0.05). The MCP-1 mRNA levels observed in the groups treated with PTL, MCL, IAL, arglabin, compound **7**, and compound **9** were lower than those of the HG group (*p* < 0.05) (Figure 4). 

**Figure 5:**
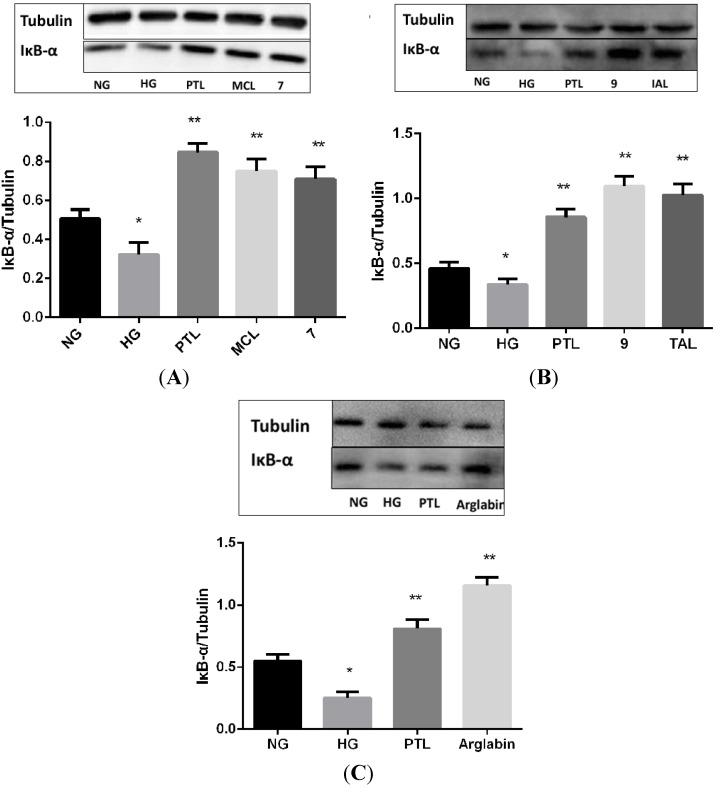
Effect of sesquiterpene lactones on HG-induced regulation of IκBα protein in MCs. NG: normal glucose; HG: high glucose. * *p* < 0.05 *vs.* NG, ** *p* < 0.05 *vs.* HG.

The level of IκBα protein was analyzed by western blotting. The degradation of IκBα was more apparent in the HG group than in the NG group (*p* < 0.05), and treatment with 10 μM of PTL, MCL, IAL, compound **7**, compound **9** and Arglabin suppressed the HG-induced degradation of IκBα (*p* < 0.05) (Figure 5).

**Figure 6:**
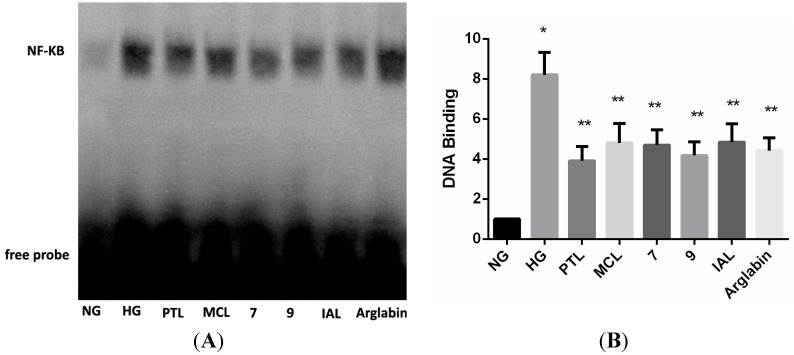
Sesquiterpene lactones inhibits high glucose-mediated NF-κB DNA binding activity. Mesangial cells treated with 10 μM of PTL, MCL, IAL, **7**, **9** and Arglabin for 1 h prior to high glucose induction were analyzed for NF-κB DNA binding activity by electrophoretic mobility shift assays (EMSA). NG: normal glucose; HG: high glucose. * *p* < 0.05 *vs.* NG, ** *p* < 0.05 *vs.* HG.

The EMSA data indicated that the NF-κB DNA binding activity was substantially enhanced in the HG group compared with the NG group when the cells were treated with 10 μM of PTL, MCL, IAL, compound **7**, compound **9** and Arglabin indicating that these treatments significantly decreased the HG-induced regulation of NF-κB DNA binding activity (Figure 6).

**Figure 7:**
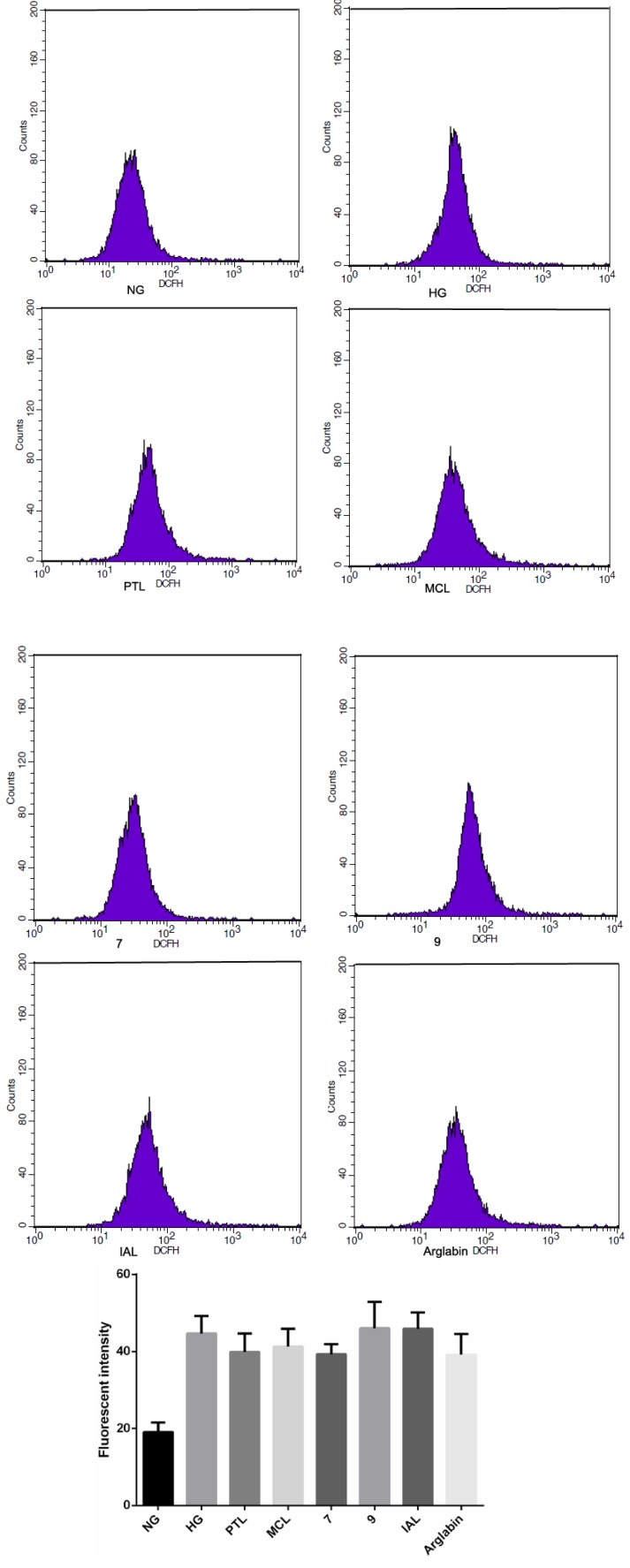
HG induces ROS production in MCs, MCs were pretreated with SLs before HG stimulation for 30 min. NG: normal glucose; HG: high glucose.

To examine the ROS-mediated signaling cascade in the MCP-1, TGF-β1 and FN production, we examined whether SLs (10 μmol/L) inhibited HG-induced ROS production (Figure 7). However, these inhibitors failed to block intracellular ROS production, suggesting that ROS production is upstream of NF-κB activation.

**Figure 8:**
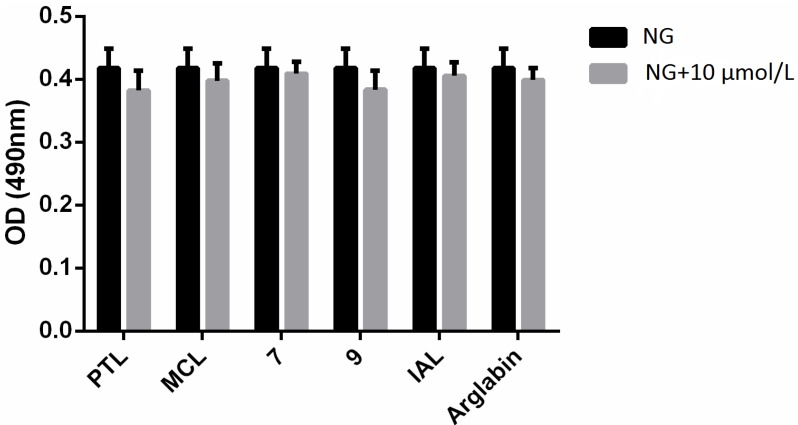
Effect of sesquiterpene lactones on viability of MCs at a concentration of 10 μM. NG: normal glucose for 24 h.

In order to evaluate the cytotoxic potential of the SLs, their effects on viability of MCs used in the proliferation assay was studied (Figure 8). Up to a concentration of 10 μmol/L, no cytotoxic effects could be observed using MTT method.

In this study, we analyzed a series of SLs (PTL, MCL, IAL, arglabin, and their analogs—compounds **7** and **9**) and found that the proliferation of MCs, the degradation of IκBα and the activation of NF-κB that were induced by a high concentration of glucose were inhibited by treatment with SLs. Researcher have previous reported that some SLs with a wide range of biological activities including anticancer and anti-inflammatory properties by inhibiting the activation of NF-κB. [25,26]. Our study revealed that the expression of MCP-1, TGF-β1 and FN were increased in the presence of an elevated glucose concentration in rat glomerular MCs and their biosynthesis and excretion were significantly inhibited in the presence of 10 μM of PTL, MCL, IAL, arglabin, compounds **7** and **9**. However, at the concentration of 0.2 and 1 μM were significantly less effective or ineffective. To the best of our knowledge, this study is the first work to show that SLs protect against DN mainly through NF-κB inhibition.

Regarding the stability of the dehydrocostus lactone, we considered that the two unconjugated *exo*-double bonds might be responsible for the polymerization of this compound. Therefore, we hypothesized that the replacement of the unconjugated *exo*-double bonds with potentially bioisosteric cyclopropyl moieties might provide a novel class of stable spirocyclopropyl dehydrocostus lactone analogs. Moreover, the cyclopropane motif is found in many natural products, and this three-membered structure has recently attracted special attention for the appealing structure and their pharmacological interests [43]; the spirocyclopropyl scaffold is indeed a useful tool for the design of constrained bioactive molecules that project pharmacophores into the appropriate protein binding pockets [44,45]. Compound **7** is bench-stable in the solid state. The stability of dehydrocostus lactone and compound **7** was also measured in simulated stomach acid (pH 1.0 at 37 °C). The half-life of compound **1** is only 0.44 h, whereas that of compound **5** is 14.6 h. This study suggested that replacement of the two *exo*-double bonds with cyclopropyl moieties has improved the stability 33-fold. The result suggested that the spirocyclopropyl dehydrocostus lactone analog (compound **7**) was substantially more stable than the naturally occurring dehydrocostus lactone as a result of the replacement of the two double bonds of the compound with two cyclopropanes.

## 3. Experimental

### 3.1. Materials

Rat glomerular MC (Wuhan University, Wuhan, China); 0.25% trypsin, DMEM, fetal bovine serum (GIBCO, GrandIsland, NY, USA); Inhibitor kappa B antibody (Cell Signaling Technology, Boston, MA, USA); MCP-1 and TGF-β1ELISA kit (eBioscience, San Diego, CA, USA); Nuclear and Cytoplasmic Protein Extraction Kit and EMSA kit (Thermo, Rockford, IL, USA), Biotin mark NF-κB probe (Beyotime Institute of Biotechnology, Haimen, China), PTL and its derivatives were provided by Accendatech Co., Ltd. (Tianjin, China).

### 3.2. Methods

*Cell culture and treatment*. Mesangial cells were incubated in low-glucose (LG) media with 10% fetal bovine serum, at 37 °C in humidified atmosphere with 5% CO_2_. When the MCs showed aggregation growth, gradually fused and passaged them, switched to serum-free low glucose DMEM and synchronized for 24 h, the experiments were divided into 20 groups: (1) normal glucose group (NG, 5.6 mM); (2) high glucose group (HG, 30 mM); (3) treated with PTL (0.2, 1, 10 μM) exposed to high glucose; (4) treated with MCL (0.2, 1, 10 μM) exposed to high glucose; (5) treated with **7** (0.2, 1, 10 μM) exposed to high glucose. (6) treated with **9** (0.2, 1, 10 μM) exposed to high glucose; (7) treated with IAL (0.2, 1, 10 μM) exposed to high glucose; (8) treated with arglabin (0.2, 1, 10 μM) exposed to high glucose.

*MTT for cell proliferation*. Took the logarithmic phase cells and put equal number of mesangial cells into cell cultured plate with the density of 1 × 10^4^ cells each hole, switched confluent MCs to serum-free low glucose DMEM and synchronized them for 24 h, then joined the high-glucose DMEM containing PTL and its derivatives, cultured for 24 h, removed the DMEM , add 0.5% MTT to each well and the cells were incubated for 4 h, then added 150 μL of DMSO to each well and put the cell cultured plate on shaking table with low speed oscillation for 10 min, the absorbance was measured at 490 nm by enzyme linked immunosorbent detector when crystallization dissolved, and the cell proliferation activity was expressed the absorption value.

*Enzyme-linked immunosorbent assay (ELISA) for MCP-1, TGF-β1 and FN*. MCs (1 × 10^5^ cells/well in 24-well plate) were preincubated with or without PTL and its derivatives in 5.6 (normal glucose, NG) or 30 mmol/L glucose (high glucose, HG) for the next 24 h. Subsequently, the supernatants were assayed for the levels of MCP-1 TGF-β1 and FN using ELISA kits, according to the manufacturer’s procedures.

*Real-time quantitative PCR for MCP-1 and TGF-β1mRNA*. After treated with PTL and its derivatives for 24 h under high glucose, total RNA was prepared from cell lines using TRIzol reagent (Takara, China) and cDNA synthesis were done with Oligo dT (Invitrogen, Shanghai, China). SYBR Premix Ex TaqTM was used for testing through Real-time fluorescent quantitative PCR instrument (FTC-2000 type, Funglyn, Toronto, Canada), housekeeping gene GAPDH was used as a reference gene in this study. The primer sequences were is as follows: MCP-1: 5'-TAGCATCCACGTGCTGTCTC-3' (forward), and 5'-CCGACTCATTGGGATCATCT-3' (reverse), and the molecular weight of amplified DNA was 91bp; TGF-β1: 5'-TGAGTGGCTGTCTTTTGACG-3' (forward), and 5'-TGGGACTGATCCCATTGATT-3' (reverse), and the molecular weight of amplified DNA was 146bp; GAPDH: 5'-ATTGTCAGCAATGCATCCTG-3' (forward), and 5'-ATGGACTGTGGTCATGAGCC-3' (reverse), and the molecular weight of amplified DNA was 102bp. Reactive conditions: 94 °C preliminary degeneration 2 min, 94 °C modified 15 s, 40 cycles at 60 °C for 30 s. The above experiment repeated three times. Through the ΔCt method for statistical analysis (the purpose of the gene expression compared with control group change ratio).

*Western blot for IκBα protein*. The logarithmic phase cells MCs (1 × 10^5^ cells/well in 10 cm petri dishes) were preincubated in 5.6 mmol/L glucose. MCs were treated with PTL and its derivatives (10 μM) for 0.5 h in high glucose when cell density growth to about 80% to 90%. Swilled MCs with precooling PBS for three times, spall MCs, samples were resolved by 10% SDS-PAGE, transferred to PVDF membranes, and blocked in 5% skim milk powder blocking buffer. The membranes were incubated with primary antibody overnight at 4 °C, followed by secondary antibody for 1 h before exposure. The quantitative analysis of these images was performed using the ImageJ and the optical density was normalized against tubulin.

*EMSA for NF-κB activity*. MCs were treated with PTL and its derivatives (10 μM) for 1 h in high glucose. MCs were rinsed with precooled PBS three times, nucleoprotein was extracted according to the manufacturer’s procedures, supernatant fluid collected after centrifugation, and the Coomassie Brilliant Blue method used for nucleoprotein quantitation. Nucleoprotein concentration was set at 1 μg/μL, and preserved in −70 °C. The NF-κB biotin mark probe sequence was 5′-AGT TGA GGG GAC TTT CCC AGG C-3′ and 3′-TCA ACT CCC CTG AAA GGG TCC G-5′. Established the probe and nucleoprotein binding reaction system, the reaction liquids were analyzed by PAGE electrophoresis, observed and photographs recorded with an ltra-violet transmission analyzer.

*Determination of intracellular ROS by flow cytometry*. The levels of intracellular ROS were determined by measuring samples with an oxidation-sensitive fluorescent probe 2',7'-dichlorofluorescein diacetate (DCFH-DA) (Sigma-Aldrich, Saint Louis, MO, USA). This compound readily diffuses into cells and is deacetylated to form nonfluorescent 2',7'-dichlorofluorescein (DCFH), which emits fluorescence when it reacts with ROS to form the highly fluorescent 2',7'-dichlorofluorescein (DCF). The cells were washed with D-Hank’s solution and then incubated with 10 μM of DCFH-DA for 30 min at 37°C. The distribution of DCF fluorescence distribution in cells was detected on a flow cytometer using a wavelength at 488 nm for excitation and 525 nm for emission.

*Statistical analysis*. Results are expressed as the mean ± standard error of the mean (SEM) form at least three independent experiments. SPSS13.0 statistical software was used for statistical analysis, statistical significance was determined using analysis of variance, a P-value of less than 0.05 was considered statistically significant.

*Stability tests of dehydrocostus lactone and compound* **7** *in simulated stomach acid*. A 100 μL portion of a 1.0 mg dehydrocostus lactone (or compound **7**) stock solution (in acetonitrile) was added to 1.0 mL of a freshly prepared 0.1 N HCl aqueous solution (preheated to 37 °C). The resulting mixture was sealed to prevent water evaporation and maintained at 37 °C in a water bath. The solution samples of dehydrocostus lactone or compound **7** were taken at time intervals of 0, 5, 15, 30, 45, 60, 120, 180, 300 and 14,400 min respectively. The samples were stored in dry ice-acetone bath and analyzed (in triplicate) as quickly as possible. Analytical HPLC was applied to measure the concentration of compounds in the solutions after different time intervals.

## 4. Conclusions

The results of this study showed that some SLs suppressed the glucose-stimulated degradation of IκBα and the subsequent activation of NF-κB and that these SLs reduced the generation of the chemokine MCP-1, TGF-β1 and FN in rat glomerular MCs. The data presented here suggest that drug discovery and development based on the SL backbone may have a high potential for the identification of drug candidates for the treatment of DN, although the precise mechanisms by which the SL compounds target DN remain to be further determined. Indeed, studies of the application of DMAMCL and DMAPT in animal models of DN are currently underway.

## References

[1] Packham D.K., Alves T.P., Dwyer J.P., Atkins R., de Zeeuw D., Cooper M., Shahinfar S., Lewis J.B., Lambers Heerspink H.J. (2012). Relative incidence of ESRD *versus* cardiovascular mortality in proteinuric type 2 diabetes and nephropathy: results from the DIAMETRIC (Diabetes Mellitus Treatment for Renal Insufficiency Consortium) database. Am. J. Kidney Dis..

[2] Yusuf S., Teo K.K., Pogue J., Dyal L., Copland I., Schumacher H., Dagenais G., The ONTARGET Investigators (2008). Telmisartan, ramipril, or both in patients at high risk for vascular events. N. Engl. J. Med..

[3] Fallahzadeh M.K., Dormanesh B., Sagheb M.M., Roozbeh J., Vessal G., Pakfetrat M., Daneshbod Y., Kamali-Sarvestani E., Lankarani K.B. (2012). Effect of addition of silymarin to renin-angiotensin system inhibitors on proteinuria in type 2 diabetic patients with overt nephropathy: A randomized, double-blind, placebo-controlled trial. Am. J. Kidney Dis..

[4] Kanwar Y.S., Wada J., Sun L., Xie P., Wallner E.I., Chen S., Chugh S., Danesh F.R. (2008). Diabetic nephropathy: mechanisms of renal disease progression. Exp. Bio. Med..

[5] Navarro-González J.F., Mora-Fernández C., de Fuentes M.M., García-Pérez J. (2011). Inflammatory molecules and pathways in the pathogenesis of diabetic nephropathy. Nat. Rev. Nephrol..

[6] Min D., Lyons J.G., Bonner J., Twigg S.M., Yue D.K., McLennan S.V. (2009). Mesangial cell-derivedfactors alter monocyte activation and function through inflammatory pathways: Possible pathogenic role in diabetic nephropathy. Am. J. Physiol. Renal. Physiol..

[7] Kumar A., Negi G., Sharma S.S. (2011). JSH-23 targets nuclear factor-kappa B and reverses various deficits in experimental diabetic neuropathy: Effect on neuroinflammation and antioxidant defence. Diabetes Obes. Metab..

[8] Ji Y., Lu G., Chen G., Huang B., Zhang X., Shen K., Wu S. (2011). Microcystin-LR induces apoptosis via NF-κB/iNOS pathway in INS-1 cells. Int. J. Mol. Sci..

[9] Pai S., Thomas R. (2008). Immune deficiency or hyperactivity-Nf-kb illuminates autoimmunity. J. Autoimmun..

[10] Palsamy P., Subramanian S. (2011). Resveratrol protects diabetic kidney by attenuating hyperglycemia-mediated oxidative stress and renal inflammatory cytokines via Nrf2-Keap1 signaling. Biophys. Acta.

[11] Wu J., Guan T.J., Zheng S., Grosjean F., Liu W., Xiong H., Gordon R., Vlassara H.,  Striker G.E., Zheng F. (2011). Inhibition of inflammation by pentosan polysulfate impedes the development and progression of severe diabetic nephropathy in aging C57B6 mice. Lab. Invest..

[12] Nogueira-Machado J.A., Volpe C.M., Veloso C.A., Chaves M.M. (2011). HMGB1, TLR and RAGE: a functional tripod that leads to diabetic inflammation. Expert. Opin. Ther. Targets.

[13] Ingaramo P.I., Ronco M.T., Francés D.E., Monti J.A., Pisani G.B., Ceballos M.P., Galleano M., Carrillo M.C., Carnovale C.E. (2011). Tumor necrosis factor alpha pathways develops liver apoptosis in type 1 diabetes Mellitus. Mol. Immunol..

[14] Giunti S., Tesch G.H., Pinach S., Burt D.J., Cooper M.E., Cavallo-Perin P., Camussi G., Gruden G. (2008). Monocyte chemoattractant protein-1 has prosclerotic effects both in a mouse model of experimental diabetes and *in vitro* in human mesangial cells. Diabetologia.

[15] Chow F.Y., Nikolic-Paterson D.J., Ma F.Y., Ozols E., Rollins B.J., Tesch G.H. (2007). Monocyte chemoattractant protein-1-induced tissue inflammation is critical for the development of renal injury but not type 2 diabetes in obese db/db mice. Diabetologia.

[16] Murphy M., Docherty N.G., Griffin B., Howlin J., McArdle E., McMahon R., Schmid H., Kretzler M., Droguett A., Mezzano S. (2008). IHG-1 amplifies TGF-beta 1 signaling and is increased in renal fibrosis. J. Am. Soc. Nephrol..

[17] Lan Y., Zhou Q., Wu Z.L. (2004). NF-kappa B involved in transcription enhancement of TGF-beta 1 induced by Ox-LDL in rat mesangial cells. Chin. Med. J. (Engl.).

[18] Chen K.H., Cheng M.L., Jing Y.H., Chiu D.T., Shiao M.S., Chen J.K. (2011). Resveratrol ameliorates metabolic disorders and muscle wasting in streptozotocin-induced diabetic rats. Am. J. Physiol. Endocrinol. Metab..

[19] Ting A.Y., Endy D. (2002). Decoding NF-KB signaling. Science.

[20] Lawrence T., Bebien M., Liu G.Y., Nizet V., Karin M. (2005). IKKalpha limits macrophage NF-kappaB activation and contribution of inflammation. Nature.

[21] Giarratana N., Penna G., Amuchastegui S., Mariani R., Daniel K.C., Adorini L. (2004). A vitamin D analog down-regulates proinflammatory chemokine production by pancreatic islets inhibiting T cell recruitment and type 1 diabetes development. J. Immunol..

[22] Vaughan S., Jat P.S. (2011). Deciphering the role of nuclear factor kappaB in cellular senescence. Aging (Albany N.Y.).

[23] Janecka A., Wyrębska A., Gach K., Fichna J., Janecki T. (2012). Natural and synthetic α-methylenelactones and α-methylenelactams with anticancer potential. Drug Discov. Today.

[24] Knight D.W. (1995). Feverfew: chemistry and biological activity. Nat. Prod. Rep..

[25] Wang W.J., Wu F., Qian Y., Cheng L.F., Shao X.T., Tong X.M., Li H. (2010). Inhibition of inflammatory factors by parthenolide in human renal mesangial cells under hyperglycemic condition. Afr. J. Biotechnol..

[26] Oka D., Nishimura K., Shiba M., Nakai Y., Arai Y., Nakayama M., Takayama H., Inoue H., Okuyama A., Nonomura N. (2007). Sesquiterpene lactone parthenolide suppresses tumor growth in a xenograft model of renal cell carcinoma by inhibiting the activation of NF-kappa B. Int. J. Cancer.

[27] Schall A., Reiser O. (2008). Synthesis of biologically active guaianolides with a trans-annulated lactone moiety. Eur. J. Org. Chem..

[28] Hussain R.F., Nouti A.M., Oliver R.T. (1993). A new approach for measurement of cytotoxicity using colorimetric assay. J. Immunol. Methods.

[29] Koch E., Klaas C.A., Rüngeler P., Castro V., Mora G., Vichnewski W., Merfort I. (2001). Anti-inflammatory effects of sesquiterpene lactones by inhibition of cytokine production and lymphocyte proliferation. Biochem. Pharm..

[30] Corona D., Díaz E., Nava J.L., Guzmán A., Barrios H., Fuentes A., Hernandez-Plata S.A., Allard J., Jankowski C.K. (2005). ^1^H, ^13^C-NMR and X-ray crystallographic studies of highly polyhalogenated derivatives of costunolide lactone. Spectrochim. Acta A.

[31] Shaikenov T.E., Adekenov S.M., Williams R.M., Prashad N., Baker F.L., Madden T.L., Newman R. (2001). Arglabin-DMA, a plant derived sesquiterpene, inhibits farnesyltransferase. Oncol. Rep..

[32] Zhangabylov N.S., Dederer L.Y., Gorbacheva L.B., VasilLeva S.V., Terekhov A.S., Adekenov S.M. (2004). Sesquiterpene lactone arglabin influences DNA synthesis in P388 leukemia cells *in vivo*. Pharm. Chem. J..

[33] Ogura M., Cordell G.A., Farnsworth N.R. (1978). Anticancer sesquiterpene lactones of *Michelia Compressa* (Magoliaceae). Phytochemistry.

[34] Jacobsson U., Kumar V., Saminathan S. (1995). Sequiterpene lactones from *Michelia Champaca*. Phytochemistry.

[35] Zhang Q., Lu Y., Ding Y., Zhai J., Ji Q., Ma W., Yang M., Fan H., Long J., Tong Z. (2012). Guaianolide sesquiterpenelactones, a source to discover agents that selectively inhibit acute myelogenous leukemia stem and progenitor cells. J. Med. Chem..

[36] Wang K.T., Liu H.T., Zhao Y.K., Chen X.G., Hu Z.D., Song Y.C., Ma X. (2000). Separation and determination of alantolactone and isoalantolactone in traditional Chinese herbs by capillary electrophoresis. Talanta.

[37] Guzman M.L., Rossi R.M., Neelakantan S., Li X., Corbett C.A., Hassane D.C., Becker M.W., Bennett J.M., Sullivan E., Lachowicz J.L. (2007). An orally bioavailable parthenolide analog selectively eradicates acute myelogenous leukemia stem and progenitor cells. Blood.

[38] Zhai J.-D., Li D., Long J., Zhang H.-L., Lin J.-P., Qiu C.-J., Zhang Q., Chen Y. (2012). Biomimetic semisynthesis of arglabin from parthenolide. J. Org. Chem..

[39] Charette A.B., Juteau H., Lebel H., Molinaro C. (1998). Enantioselective cyclopropanation of allylic alcohols with dioxaborolane ligands: scope and synthetic applications. J. Am. Chem. Soc..

[40] Matsuda H., Kageura T., Inoue Y., Morikawa T., Yoshikawa M. (2000). Absolute stereostructures and syntheses of saussureamines A, B, C, D and E, amino acid-sesquiterpene conjugates with gastroprotective effect, from the roots of *Saussurea lappa*. Tetrahedron.

[41] Alves J.C.F., Fantini E.C. (2007). Study of the inversion reaction of the lactonic fusion on eremanthine derivatives. J. Braz. Chem. Soc..

[42] Cantrell C.L., Pridgeon J.W., Fronczek F.R., Becnelb J.J. (2010). Structure–activity relationship studies on derivatives of eudesmanolides from *Inula helenium* as toxicants against *Aedes aegypti Larvae* and adults. Chem. Biodiv..

[43] Wessjohann L.A., Brandt W., Thiemann T. (2003). Biosynthesis and metabolism of cyclopropane rings in natural compounds. Chem. Rev..

[44] Nocquet P.-A., Hazelard D., Compain P. (2012). Synthesis of spirocyclopropyl γ-lactams by tandem intramolecular azetidine ring-opening/closing cascade reaction: synthetic and mechanistic aspects. Tetrahedron.

[45] Day B.W., Magarian R.A., Pento J.T., Jain P.T., Mousissian G.K., Meyer K.L. (1991). Synthesis and biological evaluation of a series of 1,1-dichloro-2,2,3-triarylcyclopropanes as pure antiestrogens. J. Med. Chem..

